# Sucrose Inclusion in Gestating and Lactating Diets of Sows Modifies the Feeding Behavior of Post-Weaning Pigs for Sweet Solutions

**DOI:** 10.3390/vetsci9050233

**Published:** 2022-05-11

**Authors:** Jaime Figueroa, Carolina Valenzuela, Sergio A. Guzmán-Pino

**Affiliations:** 1 Instituto de Ciencias Agroalimentarias, Animales y Ambientales—ICA3, Universidad de O’Higgins, San Fernando 3070000, Chile; jaime.figueroa@uoh.cl; 2 Departamento de Fomento de la Producción Animal, Facultad de Ciencias Veterinarias y Pecuarias, Universidad de Chile, Santa Rosa 11735, La Pintana 8820000, Chile; cvalenzuelav@u.uchile.cl

**Keywords:** feeding behavior, post-weaning pigs, pre-natal nutrition, sucrose, taste perception

## Abstract

Pigs display an innate preference for sweet taste compounds such as sucrose. However, the influence of sucrose supplementation into maternal diets has not been examined in pigs. We tested the hypothesis that sucrose inclusion into sows’ diets would modify the feeding behavior of post-weaning pigs for sweet and umami solutions. Twenty-two sows (85 days of gestation) were used. They randomly received a gestational and lactating diet with or without 50 g/kg of sucrose. Different sucrose and monosodium glutamate solutions were offered to the progeny to analyze different intake behavior measurements during nursery. Pigs born from treated sows presented a higher sucrose threshold than control animals (15 mM vs. 0.1 mM, *p* = 0.032) and displayed decreased sensory-motivated intake for this disaccharide (*p* < 0.023). Sucrose consumption decreased (*p* < 0.021) in pigs born from treated sows, as well as the consumption patterns for the less concentrated solutions (*p* < 0.014). The inclusion of sucrose into maternal diets (gestation and lactation) could modified pigs’ feeding behavior after weaning when offered sweet solutions, which speaks against the practicality of this supplementation in pig production systems.

## 1. Introduction

Pigs are weaned at a very early age (3–4 weeks of age) in intensive production systems, being one of the most stressful events in a pig’s life [1,2,3]. Several stressors result in reduced feed intake, poor growth rate, in addition to increased maintenance requirements and high susceptibility to enteric pathogens [2,3,4]. In order to ensure feed intake, different feed additives and highly palatable ingredients have been incorporated at the beginning of the nursery period, such as commercial flavors or milk derivatives [2]. Pigs, like other mammals, can recognize the five basic tastes: umami, sweet, bitter, sour and salty [5], related to different nutrients such as L-amino acids and peptides which evoke umami and simple carbohydrates which evoke sweetness [5,6,7]. Sugars include different types of carbohydrates, polyols and sweeteners that are recognized by the pig heterodimeric taste receptor family 1 subunits 2 and 3 (T1R2/T1R3) into the oral cavity and gastrointestinal tract of pigs [8,9]. Pigs show an innate attraction and preference for solutions of sucrose, glucose, lactose and sodium saccharin when compared against water in short-term preference tests [10,11]. The attraction is like that showed by humans, reflecting a trait that has probably evolved through years to signal highly caloric carbohydrate-rich nutrients [12,13]. Previous studies have shown that sucrose is the most preferred carbohydrate in pigs, as it is in humans, presenting the highest sweet intensity of all carbohydrates [14]. Sucrose acts as a learning enhancer through its positive oral and post-oral effects related with energy supply, generating pleasure associated to its consumption. For this reason, sucrose has been employed as an additive to improve feed intake in pigs but with contradictory results [15,16]. In fact, a prior study from our group evaluated the effects of a long-term availability to a concentrated 16% sucrose solution in addition to the commercial diet of nursery pigs, observing an elevated consumption of the sweet solution with concomitant reductions in the feed intake and weight gain of the animals [13].

Maternal feeding behaviors could be learned by the horizontal or vertical transmission of the sensory information of diets, observing that newly weaned animals benefit from their mother’s dietary experiences [17]. Several studies in pigs prove that different volatile compounds can cross the trans-placental barrier, being transmitted in small amounts from the maternal diet to the amniotic fluid and milk [18,19,20,21,22,23,24,25]. The information transferred from the mother during late gestation and lactation to their litter may contribute to the autonomous pig feeding. Thus, exposing piglets through maternal diets may result in a later preference for the sensory cues related to them, thereby positively affecting the acceptance of feeds with familiar cues before and after weaning [20,21]. However, most of the studies carried out in pigs in relation to flavor continuity have mainly focused on one component, the volatile fraction [19,22,24], i.e., the one linked to food/feed aromas (odors) perceived by the oronasal cavity. Recently, we described the maternal dietary supplementation effect of monosodium glutamate (MSG) over progeny’s intake behavior after weaning [7], observing that pigs decrease their preference thresholds for MSG and sucrose and increased the sucrose-motivated intake. Prenatal supplementation with MSG also decreased pigs’ MSG-driven intake with no effect on their consumption or consumption patterns. This previous approach suggested a possible umami–sweet taste interaction. Following that study, the present work was conducted to assess the effects of maternal inclusion of another innately preferred taste compound such as sucrose on the feeding behavior of pigs. Based on our previous results, it was hypothesized that sucrose added to sows’ gestating and lactating diets would change the perception of sweet and umami taste in post-weaning pigs, particularly decreasing the preference thresholds for sucrose and MSG solutions, confirming the influence of taste-active compounds added into maternal diets.

## 2. Materials and Methods

The experiment was conducted in an intensive swine production system located in Chile’s central zone. In general, the methodology used is like that previously described in Guzmán-Pino et al. 2019 [7].

### 2.1. Gestation and Lactation

Twenty-two sows (Landrace × Large White) and 208 pigs born from these sows were used. Based on parity number (3.2 ± 0.7), BCS (3.3 ± 0.4) and back fat thickness (9.2 ± 2.0 mm), gilts were randomly assigned to one of two treatments depending on the diets delivered one month before the estimated due date: (1) commercial gestation and lactation diets (control group; Table 1) or (2) the same commercial diets with the inclusion of 50 g/kg of sucrose (sucrose group). Diets were formulated according to NRC recommendations [26] and were offered in mash form. The amount of feed given was adjusted to sows’ BCS during gestation, and an ad libitum regime was applied during the lactation period.

### 2.2. Post-Weaning Measurements

A total of 208 pigs born from experimental sows were selected. After weaning (day 21 of life) pigs were allocated in 8 pens equally distributed per experimental group. Housing and feeding conditions as well as pre-training and training periods were the same as those previously described in Guzmán-Pino et al. 2019 [7].

Pigs were subjected to sucrose and the MSG choice test in order to determine preference thresholds for such compounds. Sucrose solutions were 0.1, 0.2, 1, 6, 12, 15, 18 and 24 mM. The MSG solutions were 1, 3, 6, 9, 18 and 27 mM. Trials comprised 2 min choice tests performed to 13 pigs’ pairs per treatment. Animals were simultaneously offered the two options: water vs. sucrose/MSG at different concentrations. Each drinker was filled with 500 mL of the respective solution, and after 2 min of exposure, the drinkers were removed and weighted to determine the remaining solution. The difference between the volume of sapid solution offered and rejected was divided by the total volume consumed, multiplied by 100, to obtain the preference percentage. All pig pairs evaluated all solution*concentration combinations. For this purpose, pairs were temporarily separated within the same pen in a specific evaluation area where they entered and exited once the test was completed. Preference thresholds and sensory-motivated intakes for sucrose/MSG were calculated as previously described [7].

To evaluate solution consumption, one drinker with the same volume aforementioned was later presented in the couple of pigs (*n* = 13). Solutions evaluated were 1, 6, 12 and 18 mM sucrose and 1, 3, 9 and 27 mM MSG. As in the previous tests to determine animals’ preferences, solution refuses were subtracted to the initial contents offered. To have an approach to the hedonic perception of animals during solution intake, consumption patterns were calculated [27]. Eight video cameras were positioned in front of each pen to record pigs’ feeding behavior during ingestion [28,29].

### 2.3. Statistical Analysis

Results of pigs’ preferences and sensory-motivated intake were contrasted to their respective neutral values through a *t*-test (SAS 9.4 proc MEANS), taking into account the effect of the treatment (control or sucrose). Consumption and consumption patterns were analyzed with ANOVA by using general linear models (SAS 9.4 proc GLM) considering the same previous factor. The experimental unit was the pig-pair, with results expressed as the average of both pigs’ data. Differences at *p* ≤ 0.050 were considered statistically significant.

## 3. Results

### 3.1. Preference Thresholds

**Figure 1:**
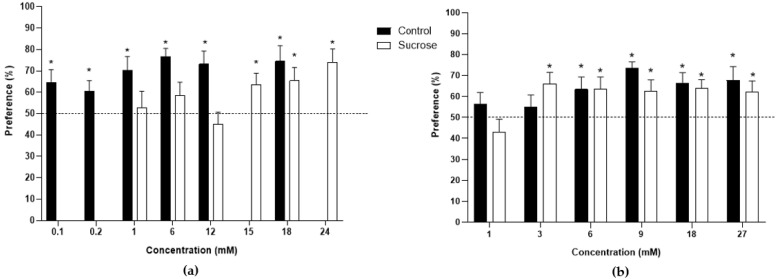
Bar plots of the preferences of pigs from control and sucrose groups: (**a**) Sucrose solutions; (**b**) MSG solutions. The dotted line represents the neutral value of 50% of preference. Asterisks highlight the values significantly (*p* < 0.05) higher than 50%.

The preference threshold for sucrose was determined at 0.1 mM in pigs born from control sows (64.6% preference, *p* = 0.032; Figure 1a). However, pigs born from sucrose-fed sows showed a preference threshold for sucrose at 15 mM (63.6% preference, *p* = 0.026). In the case of the MSG solutions, control animals displayed a significant preference in 6 mM MSG (63.4% preference, *p* = 0.040; Figure 1b). Sucrose pigs showed an MSG threshold at 3 mM (65.9% preference, *p* = 0.014).

### 3.2. Sensory-Motivated Intake

**Figure 2:**
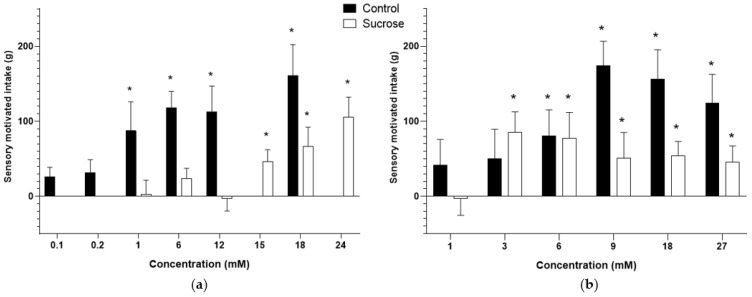
Bar plots of the sensory-motivated intake of pigs from control and sucrose groups: (**a**) Sucrose solutions; (**b**) MSG solutions. Asterisks highlight the values significantly (*p* < 0.05) higher than the negative control (no intake).

The sucrose-driven intake was detected at 1 mM in control animals (*p* < 0.043; Figure 2a). Conversely, in pigs born from sucrose-fed sows, this value was determined over 15 mM sucrose (*p* < 0.023). The MSG-motivated intake was detected in concentrations above 6 mM in control animals (*p* < 0.037; Figure 2b). In sucrose pigs, the value was determined over 3 mM MSG (*p* < 0.050).

### 3.3. Total Consumption

Sucrose consumption decreased in pigs weaned from sucrose-fed sows, as compared to animals weaned from standard sows (Table 2). This was observed by lower (*p* < 0.021) sucrose consumptions at all concentrations tested. In contrast, MSG ingestion was similar (*p* > 0.143) among control or sucrose animals.

### 3.4. Consumption Patterns

Pigs weaned from treated gilts presented lesser (*p* < 0.014) sucrose consumption patterns at 1 mM and 6 mM as compared to animals weaned from control sows (Table 3). The consumption patterns for MSG solutions were similar (*p* > 0.073) between both groups of animals.

## 4. Discussion

Several studies have previously reported the transference of flavors included in the diets of sows to their progeny [18,19,20,21,22,23,24,25]. Currently, most of the studies conducted in pigs related to flavor transference and continuity through biological fluids have focused principally on the volatile components of feeds, and little attention has been given to sapid compounds. This fact was recently showed in the review by Blavi et al. 2021 [30]. A previous work from our team displayed the effect of MSG inclusion with a similar aim [7]. Pigs show a high gustatory preference for sweet taste compounds such as sucrose [10,11,13,14]. However, to date, there is no evidence on how this performance is influenced by sucrose addition in maternal diets. For this reason, here we hypothesized that sucrose supplementation in maternal diets would modify the way that pigs behave when they are freely-offered sucrose and MSG solutions for 2 min soon after weaning.

Pigs weaned from sucrose-fed sows displayed higher preference threshold for sucrose (15 mM) compared to animals weaned from standard sows (0.1 mM), representing a 150-fold decrease in sweet sensitivity in former animals. These findings are in line with our previous study regarding MSG inclusion in maternal diets. However, it is noteworthy that the effects of sucrose inclusion were the opposite to those attributed to MSG that increased the pig sensitivity for umami. In this study, we observed a sucrose threshold much lower than 6 mM [6] in control pigs, which highlight the relevance of growing pigs as models for humans in terms of the acute detection for simple sugars from the environment. Conversely, it was observed that umami sensitivity was only one-fold apart between control and sucrose animals. Results obtained in preference thresholds evaluations were reinforced by sucrose/MSG-motivated consumption values, stating that sucrose supplementation in pigs’ maternal diets influenced both sensitivity and motivated consumption for sweetness after weaning. Another interesting finding of this study was the non-lineal increase of MSG preference and motivated consumption in control animals. In fact, the highest preference and driven intake values were registered at 9 mM MSG. Even though these concentrations also reached significant values in sucrose pigs, they were much higher in magnitude in control pigs.

When a single option was offered to pigs, it was observed that animals whose mothers were sucrose-supplemented displayed a decrease in sucrose consumption, but not in MSG consumption, relative to those animals born from control sows. It should be noted that the variation in consumption was exclusively for sucrose and not for MSG solution, which also elicits an innately preferred taste in pigs (umami) as it is sweetness. Glucose and fructose are the monosaccharide units forming sucrose. Once ingested from the diet, the absorption of monosaccharides in enterocytes is mainly mediated by Na^+^-glucose cotransporter SGLT1 and the facilitative transporters GLUT2 and GLUT5 [31,32]. During gestation, fetal glucose supply is regulated by the sow maternal body and placenta, both being important regulators in the delivery of this monosaccharide to the fetus [33]. Although the diffuse placenta present in sows is efficient in nutrients delivery [33], glucose concentration in fetal blood is much lower than that in maternal blood, entering approximately only 5% to 10% of the total, since placenta is the major consumer of glucose during gestation [34,35]. This amount, although limited, can penetrate the amniotic fluid and be perceived by the fetus by ingestion of the fluid. Hence, during late gestation (days 110–114) the fetus that ingests amniotic fluid already has a chemosensory system mature enough to detect gustatory cues dissolved in liquid phase [5,15,16]. During lactation, on the other hand, glucose and fructose ingested from the diet are utilized by the maternal organism, using about 70% of these monosaccharides to produce lactose, the major carbohydrate in milk [36,37]. In fact, it has been demonstrated that glucose inclusion in lactating sows’ diets lead to an increase in milk total solids such as lactose [38]. Even though the milk composition of control and treated sows was not analyzed in this study, it is reasonable to think that milk from sows fed sucrose during lactation supplied a higher amount of lactose to their offspring, similar to that described by Park et al. 2010 [38]. These likely changes in the nature of maternal fluids during gestation and/or lactation due to the effect of sucrose supplementation might have been perceived by the T1R2/T1R3 sweet taste receptor of pigs from the progeny and could explain the behavioral changes for sweet solutions during nursery. In this sense, it has been recognized the presence of this heterodimeric receptor at diverse tissues of the gastrointestinal tract of pigs beyond the oral cavity [5], acting as a nutrient sensor involved in the animals’ hunger–satiety cycle. Thus, the detection of monosaccharides by T1R2/T1R3 in endocrine cells triggers the release of short-term anorexigenic hormones such as CCK or GLP-1, ultimately decreasing the consumption of sapid compounds with similar sensory cues [8,9].

Behavioral changes also included modifications in the consumption patterns for sucrose, as reductions in these values were registered in this work for the less concentrated solutions (1 mM and 6 mM), which may reflect a lower hedonic perception for sweetness in comparison to control animals. Once again, the MSG-consumption patterns were not influenced by sucrose supplementation in maternal diets. An expectation for greater availability of highly-caloric carbohydrates from the environment was probably also generated in animals born from treated sows, resulting in lower recognition, lower consumption and lower pleasure perception associated with sucrose intake, as has been observed in studies of prenatal human nutrition with salt or malnourished mothers, which show that their offspring may suffer from hyponatremia and obesity, respectively, by exacerbating the seeking for salt and nutrients in postnatal life [39].

## 5. Conclusions

Pigs’ performance towards sweet sucrose solutions during nursery is affected by sucrose supplementation in maternal diets. The effects include a higher preference threshold (decreased sensitivity) and decreased sensory-motivated intake for sucrose in pigs weaned from sucrose-fed sows. In addition, sucrose supplementation in sows decreased the pigs’ intake and consumption patterns for sucrose. In relation to the practical application in intensive production systems, maternal sucrose supplementation is not advisable, as pigs become less sensitive to the detection of sweet compounds from the environment and therefore may require larger amounts to be preferred and consumed.

## Figures and Tables

**Table 1:** Maternal diets delivered.

Percentage	Gestating	Lactating
Composition		
Maize	57.2	59.4
Wheat	25.0	8.0
Soybean oil	-	4.0
Soybean meal	13.4	24.2
L-Lysine HCl	0.2	0.6
DL-Methionine	-	0.2
L-Threonine	-	0.2
L-Tryptophan	-	0.05
Mycotoxins inactivator	0.1	0.1
Mycotoxins absorbent	0.1	0.2
Artificial sweetener	0.01	-
Mineral–vitamin–phytase mix	0.4	0.4
Calcium carbonate	1.2	1.0
Sodium bicarbonate	0.7	-
Dicalcium phosphate	0.7	0.7
Salt	0.5	0.5
Copper sulphate	-	0.05
Ammonium chloride	0.3	-
Vegetable choline chloride	0.2	0.1
Chemical analysis		
DM	87.6	89.3
CP	15.4	18.7
CF	3.8	2.6
EE	2.5	5.3
NFE	60.0	56.3
Ash	5.9	6.4

**Table 2:** Consumption of pigs from control and sucrose groups for sucrose and MSG ^1^ solutions.

Solution (mM)	Consumption (g)			
	Control	Sucrose	SEM	*p*-Value
Sucrose				
1	415.9	267.2	21.60	<0.001
6	426.0	342.7	23.73	0.021
12	481.0	369.7	15.74	<0.001
18	481.3	297.4	22.15	<0.001
MSG				
1	436.7	418.7	15.44	0.410
3	472.9	448.4	12.13	0.152
9	455.4	451.4	14.01	0.833
27	445.2	479.1	16.77	0.143

^1^ Monosodium glutamate.

**Table 3:** Consumption patterns of pigs from control and sucrose groups for sucrose and MSG ^1^ solutions.

Solution (mM)	Consumption Pattern				
	Control	Sucrose	SEM	*p*-Value	
Sucrose					
1	5.1	3.8	0.35	0.014	
6	6.3	4.0	0.39	<0.001	
12	6.2	5.2	0.35	0.065	
18	6.4	5.4	0.54	0.179	
MSG					
1	5.7	6.0	0.46	0.616	
3	7.0	8.5	0.59	0.073	
9	5.6	7.0	0.58	0.090	
27	7.0	7.7	0.75	0.504	

^1^ Monosodium glutamate.

## Data Availability

The data are available upon request from the submitting author.
